# Applying Metabolomics and Aptamer-based Proteomics to Determine Pathophysiologic Differences in Decompensated Cirrhosis Patients Hospitalized with Acute Kidney Injury

**DOI:** 10.21203/rs.3.rs-4344179/v1

**Published:** 2024-05-09

**Authors:** Giuseppe Cullaro, Andrew S. Allegretti, Kavish R. Patidar, Elizabeth C. Verna, Jennifer C. Lai

**Affiliations:** University of California-San Francisco; Massachusetts General Hospital; Baylor College of Medicine and Michael E. DeBakey Veterans Affairs Medical Center; Columbia University; University of California-San Francisco

**Keywords:** Acute Kidney Injury, Proteomics, Metabolomics, Cirrhosis, Mortality, Liver Transplant

## Abstract

A case-control study of 97 patients hospitalized at our institution. We performed aptamer-based proteomics and metabolomics on serum biospecimens obtained within 72 hours of admission. We compared the proteome and metabolome by the AKI phenotype (i.e., HRS-AKI, ATN) and by AKI recovery (decrease in sCr within 0.3 mg/dL of baseline) using ANCOVA analyses adjusting for demographics and clinical characteristics. We completed Random Forest (RF) analyses to identify metabolites and proteins associated with AKI phenotype and recovery. Lasso regression models were developed to highlight metabolites and proteins could improve diagnostic accuracy. Results: ANCOVA analyses showed no metabolomic or proteomic differences by AKI phenotype while identifying differences by AKI recovery status. Our RF and Lasso analyses showed that metabolomics can improve the diagnostic accuracy of both AKI diagnosis and recovery, and aptamer-based proteomics can enhance the diagnostic accuracy of AKI recovery. Discussion: Our analyses provide novel insight into pathophysiologic pathways, highlighting the metabolomic and proteomic similarities between patients with cirrhosis with HRS-AKI and ATN while also identifying differences between those with and without AKI recovery.

## Introduction

Acute kidney injury (AKI), particularly AKI that does not reverse, is one of the deadliest complications of cirrhosis.^1–3^ Currently, the management strategies of AKI among cirrhosis patients hinge on the differential diagnosis — a broad set of pathologies ranging from functional disruptions in renal perfusion (i.e., hepatorenal syndrome (HRS-AKI)) to ischemic tubular injury (i.e., acute tubular necrosis (ATN)).^4,5^ The identification of these AKI phenotypes, particularly HRS-AKI, stems from seminal work in the 1970s and 1980s using kidney angiography that demonstrated marked renal vasoconstriction — a “functional” form of injury that could potentially reverse if kidney perfusion is restored.^6–9^

Although it is clear that kidney perfusion is markedly disrupted among patients with HRS-AKI, it is not clear how this differs from episodes of non-HRS-AKI, particularly those with ATN. For instance, contemporaneous angiography studies among patients with sepsis or ATN, demonstrated similar patterns of vasoconstriction.^10,11^ Moreover, the few pathology-based studies among patients with HRS-AKI highlight that tubular injury is exceedingly common.^12–14^ Finally, recent studies demonstrate that not only is mortality similar among those with HRS-AKI, as compared to ATN, but that similar physiologic pathways (e.g., mean arterial pressure), regardless of AKI phenotype, mediate AKI recovery.^13–15^

It is with this understanding and the intrinsic risk of kidney biopsies among cirrhosis patients that this case-control study was completed. Herein, we present the results of aptamer-based proteomic and metabolomic analyses among patients with HRS-AKI and non-HRS-AKI with and without AKI recovery. We hypothesized that although similar pathophysiologic pathways may be impacted by AKI phenotype, there would be significant differences in the proteomic and metabolomic signature by those with and without AKI recovery.

## Methods

### Population

**Figure 1:**
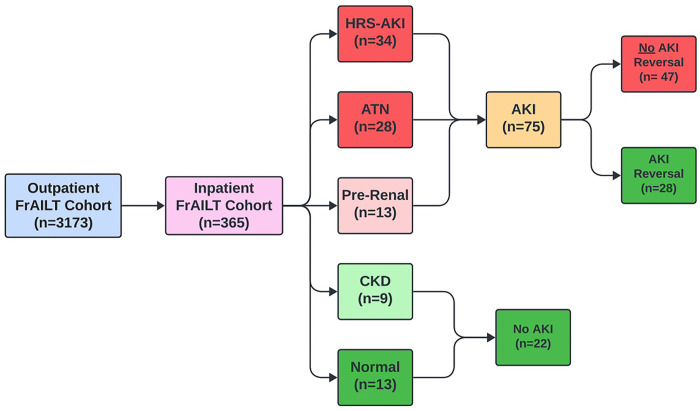
CONSORT Diagram for Case-Control Study Legend: Functional Assessment in Liver Transplantation (FrAILT); Hepatorenal Syndrome – Acute Kidney Injury (HRS-AKI); Acute Tubular Necrosis (ATN); Chronic Kidney Disease (CKD).

This study derives from an expansion of the Functional Assessment of the Study of Liver Disease (FrAILT) cohort. The FrAILT study a prospective evaluation of ambulatory patients listed for liver transplantation at the University of California, San Francisco (UCSF).^16^ Derived in 2012, the FrAILT study now encompasses > 3000 cirrhosis outpatients awaiting liver transplant. Since 2020, the FrAILT study has expanded to reevaluate these patients each time they are hospitalized at UCSF. At the time of this analysis, we had enrolled 365 hospitalized patients with decompensated cirrhosis, each with banked serum within 72 hours of their admission. Among these 365 hospitalized cirrhosis patients, we identified 34 participants with HRS-AKI, 28 participants with ATN, 13 participants with pre-renal, 9 participants with CKD, and 13 participants with normal kidney function (Fig. 1). These patients were not consecutive patients; instead, these patients were selected to ensure an adequate distribution of the types of kidney dysfunction.

### Demographics and Clinical Characterisitics

We collected the following data at the time of biospecimen collection: sex, age, race, ethnicity, etiology of cirrhosis, MELD 3.0 score^17^, ascites status, and hepatic encephalopathy status. For this study, we defined ascites as the presence of either grade 2 or grade 3 ascites. This definition was operationalized as having obtained a diagnostic paracentesis during the hospitalization.^18^ We defined hepatic encephalopathy as at least having Grade 1 (shortened attention span, impaired cognitive performance, mild confusion, or asterixis) HE in the setting of being treated with either lactulose or rifaximin.^19^

### Definition of Baseline Kidney Function

We defined baseline kidney function using a serum creatinine (sCr) 7–90 days prior to admission. Should a patient have more than 1 sCr measurement during that period, we selected the lowest value. All patients enrolled in this study had an available baseline sCr. We qualified the patient’s baseline kidney function as normal if the estimated glomerular filtration rate (eGFR) using the race-free CKD-EPI equation was ≥ 60 ml/min/1.73 m^2^ or as Chronic Kidney Disease (CKD) if the eGFR was < 60 ml/min/1.73 m^2^.^20,21^ For those without AKI at the time of biospecimen collection, we qualified them as normal or CKD based on their baseline kidney function.

### Definition of AKI, AKI Stage, and AKI Recovery

We defined AKI as a ≥ 50% increase in sCr from an outpatient baseline sCr at least 7–90 days prior to admission.

We staged AKI according to the adapted AKIN criteria from the International Club of Ascites^22–24^:

- Stage 1: a ≥ 0.3 mg/dL or ≥ 50% increase in sCr from an outpatient baseline
- ≥ Stage 2: a ≥ 200% increase from an outpatient baseline OR the initiation of kidney replacement therapy (KRT)

We defined AKI recovery as a decrease in sCr to within 0.3 mg/dL of the outpatient baseline sCr.^25^

### Definition of AKI Phenotypes

Consistent with previous studies, we defined AKI phenotypes according to the following criteria^1^:

- HRS-AKI: was defined as having the presence of ascites, a ≥ 50% increase from baseline in sCr, no response to volume expansion after 48 hours, absence of shock, no current or recent treatment with nephrotoxic drugs, absence of > 500 mg/day of proteinuria, and absence of > 50 red blood cells per high power field.
- ATN: was defined as having AKI that failed to respond to volume expansion that did not meet the diagnostic criteria for HRS-AKI, and with no current treatment with nephrotoxic drugs, absence of > 500 mg/day of proteinuria, and absence of > 50 red blood cells per high power field.
- Pre-Renal: was defined as having AKI that responded to volume expansion with a preceding clinical history consistent with ischemic or nephrotoxic AKI.

These diagnoses were confirmed through an individual chart review for each subject. Should diagnostics tests not be available (e.g., urinalysis because of oliguria), then we utilized the clinical documentation (i.e., nephrology notes) to confirm the leading diagnosis at the time of biospecimen collection and at the time of discharge.

### Biospecimens Collection and Storage

All samples were obtained within 72 hours of their admission. We used only serum samples for these analyses. All specimens were collected in serum separator tubes and processed, and the serum was stored at −80 C until the time of these analyses.

### Aptamer-based Proteomics

Sample preparation and data generation was performed by SomaLogic Inc. Samples were maintained at − 80°C until processed. For these analyses, we used the custom 1500 panel using the SomaScan system. This system uses 1500 customized aptamers, molecules designed to bind to specific proteins in body fluids (i.e., serum), offering a broad protein activity profile. Each aptamer alters its properties upon binding to its target protein, enabling detection and quantification via the high-throughput SomaScan platform. This provides a snapshot of the ‘proteome,’ or the entirety of proteins present. We include the 1500 proteins tested in **Supplemental Table 1**. For all analyses, we used log10 transformed values.

### Metabolomics

Sample preparation and data generation was performed by Metabolon Inc. Samples were maintained at − 80°C until processed. Proteins were precipitated with methanol (Glen Mills GenoGrinder 2000; Glen Mills Inc, Clifton, NJ) followed by centrifugation to remove protein, dissociate small molecules bound to protein or trapped in the precipitated protein matrix, and to recover chemically diverse metabolites. The extract is segmented into five fractions and analyzed using different UPLC-MS/MS techniques on a high-resolution mass spectrometer. The covered scan range is approximately 70–1000 m/z. Identification of compounds is achieved by comparing retention index, accurate mass, and MS/MS spectrum with Metabolon’s proprietary library. The process considers peak quantities through the area under the curve, adjusts for variance, and scales for more analysis. Missing values, believed to be below the detection limit, are imputed to the observed biochemical minimum. For all analyses, we used the natural log-transformed data. We chose statistical methodologies to align with previous metabolomic studies among cirrhosis patients.^26,27^

### Qualitatively Compare the Ability of Metabolomics and Aptamer-Based Proteomics to Distinguish between Kidney Diagnoses and AKI Recovery

Principal Component Analysis (PCA) applied to aptamer-based proteins and metabolites reduced multidimensional data into fewer dimensions (principal components), improving analysis by highlighting significant patterns. PCs (PC1 and PC2) helped in visualizing the separation between groups (e.g., normal, CKD, Pre-Renal, ATN, HRS-AKI) in a 2D PCA plot. Variations in the plot represent differences in the protein/metabolite profiles of each kidney function group. PC1 represents the maximum variance in the dataset, while PC2 captures the second most variance, orthogonal to PC1. We generated confidence ellipses to incorporate 75% of the data points for each group.

### Comparing Aptamer-based Protein and Metabolite Values by AKI Phenotype and AKI Recovery

We performed an analysis of covariance (ANCOVA) to compare metabolite and aptamer-based protein levels between those with HRS-AKI or ATN and between those with and without AKI recovery. Covariates included MELD 3.0, age, sex, baseline sCr, AKI stage, ascites status, and hepatic encephalopathy status. The results were cross-checked for significance, and metabolites and proteins with a p < 0.05 and a q < 0.05 were considered statistically significant. The q-value, as generated by Storey and Tibshirani, is a way to estimate the proportion of false positives you would get if you consider a particular result or anything more extreme as significant within a large set of tests.^28^ We demonstrate the natural log and log10 mean difference in metabolites and proteins, respectively, between groups in volcano plots.

### Identification of Key Metabolites and Proteins to Distinguish between Kidney Diagnoses and AKI Recovery

In the Random Forest (RF) analysis, each decision tree generated its predictions and corresponding out-of-bag (OOB) error rate. We optimized the number of variables tried at each split by conducting a grid search leveraging random forests’ out-of-bag error, training multiple models on different subsets of the data until no significant improvement (defined as at least 0.01) was observed in out-of-bag error rates. We optimized the number of trees in the random forest model from 50 to 1000 in steps of 50 while keeping the number of variables tried at each split constant at the optimized value, choosing the value with the lowest out-of-bag error rate. These rates served as an internal validation mechanism to ensure the model’s robustness. RF then ranked each metabolite or protein based on their “importance”, calculated through Mean Decrease Accuracy methods. The 30 metabolites or proteins with the highest importance scores were identified as the most significant. These were the features that contributed most substantially to differentiating between AKI phenotype (i.e., HRS-AKI v. ATN) and AKI recovery and are reported.

### Determining the Added Benefit of Key Metabolites and Proteins to Distinguish between Kidney Diagnoses and AKI Recovery

We developed a clinical model using predefined clinical variables associated with kidney diagnosis and AKI recovery. The variables included in the clinical model were age, sex, AKI stage, Baseline sCr, MELD 3.0 score, ascites status, and hepatic encephalopathy status. We completed logistic regression for each outcome, and model performance was evaluated by calculating the Area Under the Receiver Operating Characteristic Curve (AUC).

To select the top 30 metabolites and proteins to include in the combined models, Lasso (Least Absolute Shrinkage and Selection Operator) regression with either the top 30 metabolites alone or the top 30 proteins alone was completed. We only included the key metabolites or proteins identified through the RF analyses. Lasso regression reduced the complexity of the model by penalizing the absolute size of coefficients, effectively turning some of them to zero. We selected the optimal lambda (penalty term) that minimized the mean cross-validated error. This quality allows Lasso to perform feature selection, making it suitable for high-dimensional datasets.

We then completed logistic regression combining our clinical variables (i.e., age, sex, AKI stage, Baseline sCr, MELD 3.0 score, ascites status, and hepatic encephalopathy status) and the identified metabolites or proteins from the RF and Lasso regression models. We calculated the AUC, and the AUCs for the combined model and the clinical-only model were then compared to determine the added predictive benefit of including metabolites or proteins to predict either AKI Recovery or AKI phenotype (i.e., HRS-AKI or ATN).^29^

### Determining the Biological Function of Selected Metabolites and Proteins

We determined the biological function of metabolites and proteins that were significantly associated with our outcomes by cross-referencing the protein or metabolite IDs with appropriate databases.

### Software

Analyses were completed in R version 4.3.1 (Beagle Scouts) and R Studio using the following additional packages: ‘car’, ‘flextable’, ‘glmnet’, ‘gtsummary’, ‘ilrba’, ‘MSnSet.utils’, ‘proc’, ‘randomForest’, ‘SomaDataIO’, ‘qvalue’.

### Institutional Review Board

This study was approved by the IRB at the University of California, San Francisco.

## RESULTS

### Sociodemographics and Clinical Characteristics

As described, we identified 34 participants with HRS-AKI, 28 participants with ATN, 13 participants with pre-renal, 9 participants with CKD, and 13 participants with normal kidney function (Fig. 1). The baseline sCrs were: Normal − 0.89 mg/dL (0.67–1.09), CKD − 1.80 mg/dL (1.50–2.60), Pre-Renal − 0.90 mg/dL (0.70–1.16), HRS-AKI − 1.00 mg/dL (0.76–1.38), and ATN − 1.05 mg/dL (0.84–1.30). The sCrs at the time of biospecimen collection were: Normal − 0.91 mg/dL (0.71–1.11), CKD mg/dL − 1.46 (1.37–1.68), Pre-Renal − 1.59 mg/dL (1.25–1.75), HRS-AKI − 2.73 mg/dL (2.40–3.98), and ATN − 2.37 mg/dL (1.82–3.44). These patients had the following MELD 3.0 scores: Normal − 27 (21–28), CKD − 27 (18–28), Pre-Renal − 27 (25–31), HRS-AKI − 36 (32–40), and ATN − 34 (31–38). Participant demographic and clinical characteristics by kidney function type are demonstrated in Table 1.

### Qualitatively Compare the Ability of Metabolomics and Aptamer-Based Proteomics to Distinguish-between Kidney Diagnoses and between AKI Recovery

**Figure 2:**
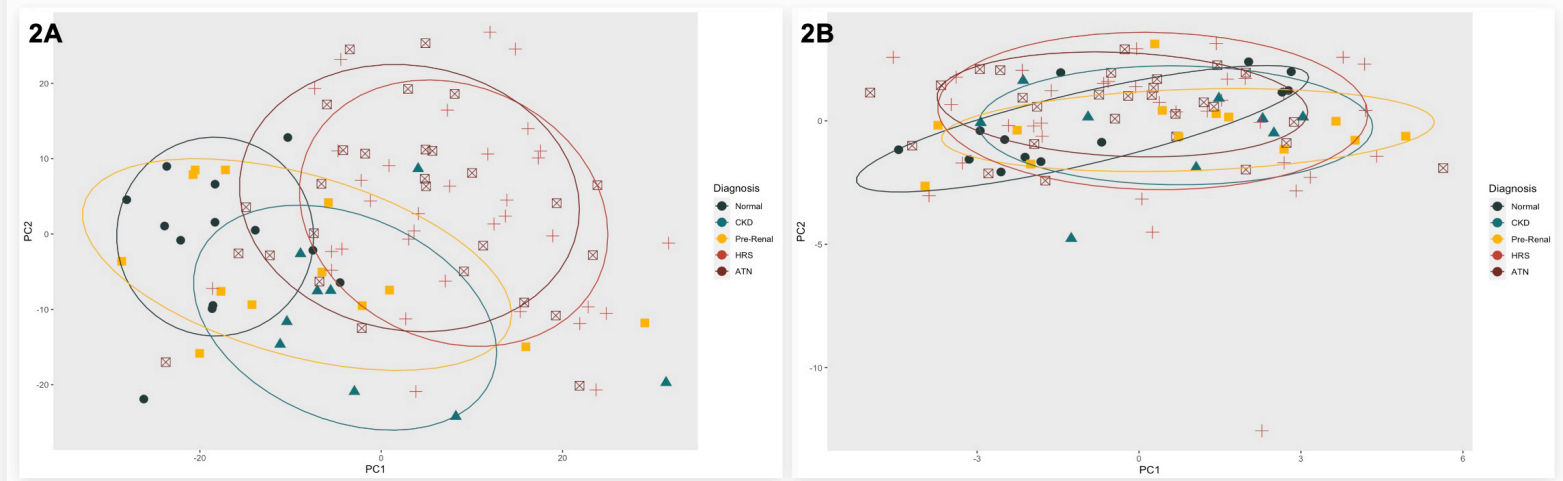
a and b. Principal Component Plots for Metabolites (A) and Proteins (B) by Kidney Function Type Legend: Hepatorenal Syndrome – Acute Kidney Injury (HRS-AKI); Acute Tubular Necrosis (ATN); Chronic Kidney Disease (CKD). * Ellipse denotes distribution of 75% of assigned group

**Figure 3:**
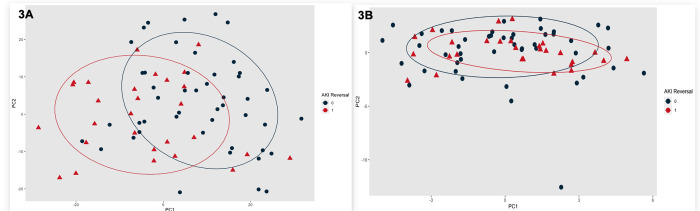
a and b. Principal Component Plots for Metabolites (A) and Proteins (B) by AKI Reversal * Ellipse denotes distribution of 75% of assigned group

We first completed a principal component analysis to qualitatively describe the metabolites and aptamer-based proteins by both kidney diagnosis and by AKI recovery status. In Figs. 2a and 2b, we depict the PC plots of the metabolites and proteins by kidney diagnosis, respectively. In Figs. 3a and 3b, we depict the PC plots of the metabolites and proteins by AKI recovery, respectively. Qualitatively, these data demonstrate that the metabolomic analyses were able to discriminate between those with and without AKI recovery. Metabolomic analyses were able to cluster for those without AKI and Pre-Renal, but there was substantial overlap in those with HRS-AKI and ATN. Our principal component analyses demonstrated that aptamer-based proteomics was not able to cluster patients either by kidney diagnosis or by AKI recovery status.

### Comparison of Proteins and Metabolites that are Most Different between Kidney Diagnoses

**Figure 4:**
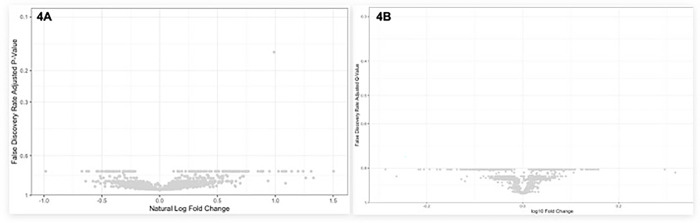
a and b. Volcano Plots for Metabolites (A) and Proteins (B) between those with HRS-AKI and ATN.

Among 62 participants with either HRS-AKI or ATN, we completed ANCOVA analyses to compare metabolite and aptamer-based protein levels. Covariates included MELD 3.0, age, sex, baseline sCr, AKI stage, ascites status, and hepatic encephalopathy status. In adjusted analyses, there were no metabolites or proteins that were significantly different between those who had HRS-AKI and those who had ATN (Fig. 4a and 4b).

### Comparison of Proteins and Metabolites that are Most Different between AKI Recovery

**Figure 5:**
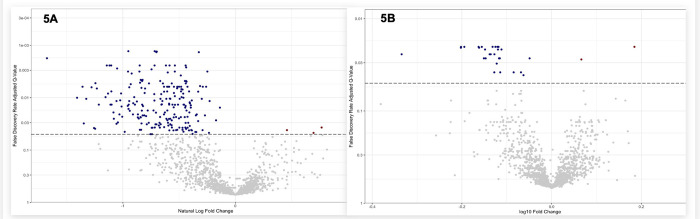
a and b. Volcano Plots for Metabolites (A) and Proteins (B) between those with and without AKI reversal.

Among the 75 participants with AKI, we compared metabolite and aptamer-based protein levels using ANCOVA analyses (Fig. 5a and 5b). Covariates included MELD 3.0, age, sex, baseline sCr, AKI stage, ascites status, and hepatic encephalopathy status. There were 217 metabolites that were significantly different, with a q-value < 0.05. These included 67 amino acid metabolites, 25 xenobiotics, 22 Lipid Metabolites, and 11 cofactors and vitamins. We have summarized these in **Supplemental Table 2**. There were 30 proteins that were significantly different, with a q-value < 0.05. Of the 30 proteins that were significantly different between the two groups, the majority included proteins that were either linked to inflammation (e.g., Tumor necrosis factor ligand superfamily member 15, Fc receptor-like protein 4:Extracellular domain), baseline kidney health (e.g., cystatin C, Beta-2 microglobulin, and Trefoil factor 3), or lipid metabolism (e.g., cholesteryl ester transfer protein). The significant proteins with the effect size, p-values, q-values, and protein targets are demonstrated in **Supplemental Table 2**.

### Identification of Key Metabolites and Proteins to Distinguish between Kidney Diagnoses

To identify the metabolites and proteins that best differentiated between HRS-AKI and ATN among the 62 patients with either diagnosis, we completed separate (i.e., one with metabolites, one with proteins) random forest models. For the metabolite model, we found that in a model with 500 trees, with 39 variables tried at each split, the OOB estimate of the error rate was 48%. We included the top 30 metabolites in **Supplemental Table 3**. For the protein model, we found that in a model with 50 trees, with 38 variables tried at each split, the OOB estimate of the error rate was 50%. We include the top 30 proteins identified in **Supplemental Table 3**.

### LASSO Regression to Determine Added Benefit of Proteomics and Metabolomics to Improve Kidney Diagnoses

Among the 62 participants with either HRS-AKI or ATN, the base clinical model demonstrated an AUC of 0.76 (95% CI: 0.63–0.88). The inclusion of the selected metabolites significantly increased the AUC for a model to diagnose HRS-AKI to 0.97 (95% CI 0.93–0.99) (p < 0.001) (Table 2). Our Lasso regression model, incorporating the top 30 proteins identified through the RF models, found that none of these proteins were significantly associated with AKI diagnosis; we, therefore, did not build a Clinical and Protein Model to diagnose HRS-AKI (Table 2).

### Identification of Key Metabolites and Proteins to Distinguish between AKI Recovery

To identify the metabolites and proteins that best differentiated between those with and without AKI recovery among the 75 patients with AKI, we completed separate (i.e., metabolite only, protein only) random forest models. For the metabolite model, we found that in a model with 500 trees, with 39 variables tried at each split, the OOB estimate of the error rate was 29%. We include the top 30 metabolites identified in **Supplemental Table 4**. For the protein model, we found that in a model with 50 trees, with 38 variables tried at each split, the OOB estimate of the error rate was 31%. We include the top 30 proteins identified in **Supplemental Table 4**.

### LASSO Regression to Determine Added Benefit of Proteomics and Metabolomics to Improve AKI Recovery

Among the 75 participants with AKI, the base clinical model demonstrated an AUC of 0.82 (95% CI: 0.73–0.92) to predict AKI recovery. The inclusion of the selected metabolites significantly increased the AUC for a model to predict AKI recovery to 0.94 (95% CI 0.89–0.99) (p = 0.01) (Table 2). The inclusion of the selected proteins increased the AUC for a model to predict AKI recovery to 0.95 (95% CI: 0.91–0.99) (p = 0.02) (Table 2).

## Discussion

Limited by the intrinsic risk of kidney biopsies among cirrhosis patients, we applied unbiased techniques — aptamer-based proteomics and metabolomics — to inform the pathophysiologic pathways that are either up or down-regulated by kidney diagnosis and AKI recovery. We highlight in ANCOVA analyses, adjusted for clinical covariates and the false discovery rate, that there were no metabolomic or aptamer-based proteomic differences between those with HRS-AKI and ATN while identifying significant differences between those with and without AKI recovery. Our random forest and Lasso regression analyses demonstrated that metabolomics can improve the diagnostic accuracy of both AKI diagnosis and AKI recovery, and aptamer-based proteomics can improve the diagnostic accuracy of AKI recovery. Our analyses provide novel insight into pathophysiologic pathways, highlighting the metabolomic and proteomic *similarities* between patients with cirrhosis with HRS-AKI and ATN while also identifying *differences* between those with and without AKI recovery.

While our principal component analyses revealed metabolomic clusters that distinguished those with HRS-AKI or ATN from those with normal kidney function, CKD, and Pre-Renal AKI, there was substantial overlap between those with HRS-AKI and ATN. We demonstrate this overlap further in our ANCOVA analyses, where when adjusted for clinical covariates and the false discovery and testing for over 1500 metabolites, there were no significant differences. Our random forest and Lasso regression models highlight that metabolites can improve the diagnostic accuracy of clinical variables alone, with metabolites associated with Methionine, Cysteine, SAM and Taurine Metabolism, Glycolysis, Gluconeogenesis, and Pyruvate Metabolism, Xanthine Metabolism, Glutamate Metabolism, and Xenobiotics being most associated with an HRS-AKI diagnosis as compared to an ATN diagnosis — findings that need to be externally validated.

Unlike the metabolomic analyses, our aptamer-based proteomic analysis did not appear to distinguish between any of the kidney function phenotypes. These results were surprising, as this panel included proteins that had previously been associated with both AKI phenotypes and AKI recovery among cirrhosis patients (e.g., neutrophil gelatinase-associated lipocalin [NGAL], kidney injury molecule-1).^30–33^ One possibility for this discordance was the utilization of serum as opposed to urine for this study. This was an intentional decision, as oliguria is common among patients with HRS-AKI, one which impacts the feasibility of completing these measurements, as well as the future implementation of any findings. That said, even though previous studies have demonstrated that *urine* NGAL levels are more strongly associated with outcomes than *serum* NGAL levels, there still appears to be a diagnostic utility to *serum* NGAL levels.^34^ A second possibility is the nature of this case-control study. These were sick, decompensated cirrhosis patients who were hospitalized — as such, even those without or with mild kidney dysfunction could have concomitant medical issues that would increase or decrease particular biomarkers (e.g., acute-on-chronic liver failure and NGAL).^35^ Collectively, each of our analyses—principal component, ANCOVA analyses, and random forests/Lasso regression—demonstrated that aptamer-based proteomics did not significantly identify patients with HRS-AKI as opposed to those with ATN—a finding that suggests that the protein response to HRS-AKI and ATN are similar.

Both metabolomics and aptamer-based proteomics were able to clearly differentiate between those with and without AKI Recovery. In the metabolomic analyses, we identified 217 metabolites that were significantly different between the two groups — metabolites involved in purine metabolism, amino acid metabolism, lipid metabolism, and xenobiotics; these metabolites encompassed several pathophysiologic pathways, demonstrating the comprehensive physiologic disarray of AKI among decompensated cirrhosis patients. The aptamer-based proteomic analyses corroborated these findings. Of the 30 proteins that were significantly different between the two groups, the majority included proteins that were either linked to inflammation (e.g., Tumor necrosis factor ligand superfamily member 15, Fc receptor-like protein 4:Extracellular domain), baseline kidney health (e.g., cystatin C, Beta-2 microglobulin, and Trefoil factor 3), or lipid metabolism (e.g., cholesteryl ester transfer protein). Collectively these analyses, in a simplified conclusion, highlight that AKI recovery is determined by the degree of inflammation or injury and the baseline kidney health.

This study has several limitations. First, our sample size. We accounted for this sample size statistically by adjusting for the false discovery rate using stringent criteria (i.e., q-values < 0.05) and attempted to limit overfitting with more advanced techniques (i.e., random forests through bagging and random subspace methods to reduce the variance, lasso regression by adding a penalty term to shrink non-informative coefficients to zero). However, the possibility of false negatives leading to limited findings, particularly between those with HRS-AKI and ATN, is possible. That said, this represents the first investigation of metabolomics and aptamer-based proteomics among hospitalized cirrhosis patients with AKI, and even with the sample size, significant differences between those with and without AKI recovery were found. Second, AKI recovery is a dynamic process, and for this study, proteomics and metabolomics were completed only at a single time point. Our cohort and biorepository have been expanded. Future work will center on repeated measures and changes in these pathophysiologic processes that dictate AKI recovery. Finally, hospitalized patients with decompensated cirrhosis are complex, which makes the identification of key pathophysiologic pathways difficult, as there are many opportunities for confounding.^36–38^ We address this in this study through our case-control design and sample size, which allowed for the review of each medical record to confirm the diagnosis and account for a substantial amount of confounding; nevertheless, the possibility of residual confounding remains.

Despite these limitations, our study applies unbiased methodologies (e.g., metabolomics, aptamer-based proteomics) to determine what pathophysiologic differences exist between those with HRS-AKI and ATN and those with and without AKI recovery. For the former, our data suggest that these clinical diagnoses may be more pathophysiologically similar than appreciated in practice — a novel finding that, if validated, would have important implications for clinical practice. For the latter, our data highlight key differences in both the metabolome and the aptamer-based proteome that inform the pathophysiologic mechanisms of AKI recovery. Collectively, we believe these analyses provide a “proof-of-concept” for the utilization of unbiased multi-omics to inform the pathophysiology of AKI among decompensated cirrhosis patients.

## Figures and Tables

**Table 1:** Characteristics by Kidney Diagnosis at Time of Biospecimen Collection.

Characteristic	Normal, N = 13^1^	CKD, N = 9^1^	Pre-Renal, N = 13^1^	HRS, N = 34^1^	ATN, N = 28^1^	p-value^2^
sCr at Biospecimen Collection	0.91 (0.71, 1.11)	1.46 (1.37, 1.68)	1.59 (1.25, 1.75)	2.73 (2.40, 3.98)	2.37 (1.82, 3.44)	< 0.001
sCr at Outpatient Baseline	0.89 (0.67, 1.09)	1.80 (1.50, 2.60)	0.90 (0.70, 1.16)	1.00 (0.76, 1.38)	1.05 (0.84, 1.30)	< 0.001
Age (Years)	59 (52, 62)	65 (62, 67)	55 (51, 63)	59 (53, 65)	56 (49, 68)	0.4
Sex	4 (31%)	5 (56%)	4 (31%)	19 (56%)	11 (39%)	0.4
Race						0.082
Black	0 (0%)	0 (0%)	0 (0%)	0 (0%)	2 (7.1%)	
Other	0 (0%)	2 (22%)	2 (15%)	1 (2.9%)	5 (18%)	
White	13 (100%)	7 (78%)	11 (85%)	33 (97%)	21 (75%)	
Ethnicity						0.3
Hispanic	5 (38%)	1 (11%)	4 (31%)	16 (47%)	13 (46%)	
Non-Hispanic	8 (62%)	8 (89%)	9 (69%)	18 (53%)	15 (54%)	
Etiology						
ARLD	7 (54%)	4 (44%)	9 (69%)	18 (53%)	9 (32%)	
Other	2 (15%)	0 (0%)	0 (0%)	3 (8.8%)	2 (7.1%)	
SLD	1 (7.7%)	3 (33%)	0 (0%)	11 (32%)	10 (36%)	
Viral Hepatitis	3 (23%)	2 (22%)	4 (31%)	2 (5.9%)	7 (25%)	
MELD 3.0 at Biospecimen Collection	27 (21, 28)	27 (18, 28)	27 (25, 31)	36 (32, 40)	34 (31, 38)	< 0.001
Hepatic Encephalopathy						0.4
None	8 (62%)	2 (22%)	8 (62%)	18 (53%)	16 (57%)	
Present	5 (38%)	7 (78%)	5 (38%)	16 (47%)	12 (43%)	
Ascites						0.054
Grade 0/1	9 (69%)	5 (56%)	4 (31%)	13 (38%)	19 (68%)	
Grade 2/3	4 (31%)	4 (44%)	9 (69%)	21 (62%)	9 (32%)	
AKI Stage						
No AKI	13 (100%)	9 (100%)	0 (0%)	0 (0%)	0 (0%)	
Stage 1 AKI	0 (0%)	0 (0%)	11 (85%)	3 (8.8%)	9 (32%)	
≥ Stage 2 AKI	0 (0%)	0 (0%)	2 (15%)	31 (91%)	19 (68%)	
Full AKI Reversal	-	-	12 (92%)	4 (12%)	12 (43%)	< 0.001

1 Median (IQR); n (%)

2 Kruskal-Wallis rank sum test; Fisher’s exact test

**Table 2.** AUC of Base Clinical Model and Models with Metabolites and Proteins for AKI Diagnosis and AKI Reversal

**Models to determine AKI Phenotype**					
**Clinical**	**AUC (95CI)**	Clinical and Metabolites*	**AUC (95CI)**	Clinical and Proteins*	**AUC (95CI)**
Sex	0.76 (0.63 – 0.88	N-acetylputrescine	0.97^#^ (0.93 – 0.99)	-	-
Age		Cysteinylglycine			
MELD 3.0		Methionine			
AKI Stage		Betaine			
Baseline sCr		X-12714			
Presence of Hepatic Encephalopathy		X-17328			
Presence of Ascites		X.19438			
**Models to determine AKI Recovery**					
**Clinical**	**AUC (95CI)**	Clinical and Metabolites*	**AUC (95CI)**	Clinical and Proteins*	**AUC (95CI)**
Sex	0.82 (0.73 – 0.92)	Pyroglutamylvaline	0.94^#^ (0.89 – 0.99)	Induced myeloid leukemia cell differentiation protein Mcl-1	0.95^#^ (0.91 – 0.99)
Age		N1-methyl-2-pyridone-5-carboxamide		Beta-1,4-galactosyltransferase 1	
MELD 3.0		N6-acetyllysine		Collagen alpha-2(VI) chain	
AKI Stage		N,N,N-trimethyl-alanylproline betaine (TMAP)		Protein FAM49B	
Baseline sCr		N-lactoyl valine		Endothelial cell-selective adhesion molecule	
Presence of Hepatic Encephalopathy		X-16938		Calcitonin receptor	
Presence of Ascites		X-17343		Protein Wnt-10b	
		X-25983		Interleukin-10 receptor subunit beta	
				Platelet-activating factor acetylhydrolase IB subunit beta	
				Insulin-like growth factor-binding protein 6	

* indicates model adjusted for Clinical Variables and all metabolites/proteins listed

# indicates <0.05 compared to clinical model alone

## Data Availability

These data contain PHI and has not been uploaded for public use. These data can be shared but a data use agreement will be needed.
